# A Hardware-Supported Algorithm for Self-Managed and Choreographed Task Execution in Sensor Networks

**DOI:** 10.3390/s18030812

**Published:** 2018-03-07

**Authors:** Borja Bordel, Carlos Miguel, Ramón Alcarria, Tomás Robles

**Affiliations:** 1 Departamento de Ingeniería de Sistemas Telemáticos, Universidad Politécnica de Madrid, Avenida Complutense No. 30, 28040 Madrid, Spain; carlos.miguel@upm.es (C.M.); tomas.robles@upm.es (T.R.); 2 Departamento de Ingeniería Topográfica y Cartografía, Universidad Politécnica de Madrid, UPM Campus Sur, Km 7.5 de la Autovía de Valencia, 28031 Madrid, Spain; ramon.alcarria@upm.es

**Keywords:** Wireless Sensor Networks, task execution, algorithms, resource management, ModelSim

## Abstract

Nowadays, sensor networks are composed of a great number of tiny resource-constraint nodes, whose management is increasingly more complex. In fact, although collaborative or choreographic task execution schemes are which fit in the most perfect way with the nature of sensor networks, they are rarely implemented because of the high resource consumption of these algorithms (especially if networks include many resource-constrained devices). On the contrary, hierarchical networks are usually designed, in whose cusp it is included a heavy orchestrator with a remarkable processing power, being able to implement any necessary management solution. However, although this orchestration approach solves most practical management problems of sensor networks, a great amount of the operation time is wasted while nodes request the orchestrator to address a conflict and they obtain the required instructions to operate. Therefore, in this paper it is proposed a new mechanism for self-managed and choreographed task execution in sensor networks. The proposed solution considers only a lightweight gateway instead of traditional heavy orchestrators and a hardware-supported algorithm, which consume a negligible amount of resources in sensor nodes. The gateway avoids the congestion of the entire sensor network and the hardware-supported algorithm enables a choreographed task execution scheme, so no particular node is overloaded. The performance of the proposed solution is evaluated through numerical and electronic ModelSim-based simulations.

## 1. Introduction

Wireless Sensor Networks (WSN) have evolved from a theoretical and research paradigm to be a real and practical technology. Actually, several new engineered systems such as Cyber-Physical Systems [1], Smart cities [2] or Industry 4.0 [3] (among other possibilities) are based on WSN. Currently, besides, WSN have been generalized [4], so they are now understood as sets of wireless smart nodes (including each one, sensors, actuators and processing devices) networked via radio links. With this new approach, WSN can not only provide information (which must be compressed and transmitted to user applications) as traditionally but also execute and get the result of tasks which are delegated by high-level applications.

First real WSN were composed of a small and homogeneous collection of nodes with a medium size [5]. Nodes in these first deployments had also a mid-range computing power, with enough resources to execute both data and control processes. However, over time, nodes became smarter (they implement more complex algorithms), smaller (thanks to microelectronic technology) and more resource-constraint (they are dedicated devices and have a lower cost) [6]. At the same time, the number of nodes in a WSN has gone up strongly and quickly; so WSN evolved to current pervasive sensing platforms composed of a huge number of tiny, heterogeneous, resource-constraint smart nodes [7].

In this context, collaborative or choreographed task execution schemes are which fit in the most perfect way with the nature of sensor networks, as they enable the creation of flexible ad hoc resource pools being able to execute delegated tasks [8]. The flexible and dynamic combination of nodes with different capabilities is the perfect approach to make the most of the resources of a sensor network, as resources are packed and assigned to tasks at real-time depending on the current node situation and workload. Nevertheless, although these solutions were investigated in first WSN, current software solutions for choreographed task execution and resource self-management by the smart nodes in a WSN are pretty inefficient: they are not adapted to resource constraint devices and resource consumption of these algorithms grows strongly with the number of nodes in the network.

**Figure 1:**
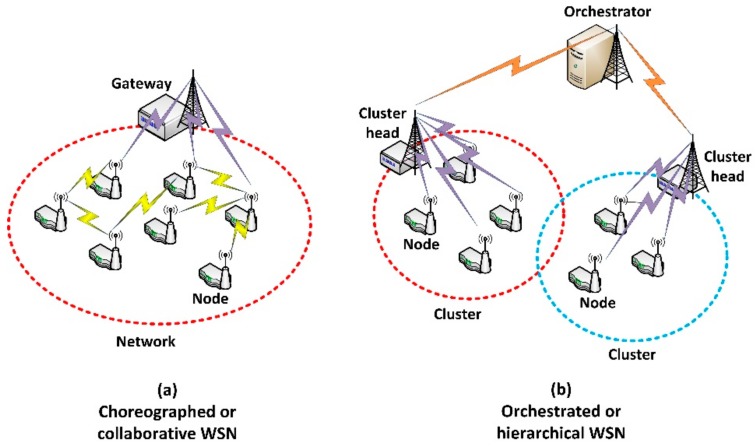
(**a**) Choreographed Wireless Sensor Network (WSN) (**b**) Hierarchical WSN.

On the contrary (see Figure 1), almost every current WSN is based on a hierarchical network design, where resource constraint nodes are grouped in clusters which are managed by more powerful computing devices usually named as “cluster heads” [9]. These cluster heads are, finally, controlled by a central orchestrator, placed in the cusp of the network hierarchy. Both, cluster heads and orchestrator present enough computing power to support any required management algorithm or solution. However, although this approach solves many management problems of large WSN with resource constraint devices, it is also very inefficient, whereas a relevant percentage of the operation time is wasted in communicating nodes with the cluster heads and the orchestrator [10]. In fact, if a conflict occurs, nodes must request cluster heads and orchestrator for a solution and wait for instructions before continuing with the operation. Next-generation WSN, applied to Industry 4.0 for example, must operate at real-time, so new and more efficient management solutions are required.

Therefore, in this paper it is proposed a new solution for self-managed and choreographed task execution in sensor networks, which is adapted to resource-constraint devices and may be implemented in large WSN. This proposal is based on a horizontal network scheme where they are not required cluster heads or heavy orchestrators. Besides, the workload that sensor networks support is improved, as almost no time is wasted in communicating nodes with other components during control transactions. Thus, efficiency is also improved. These objectives are reached thanks to choreography and self-management algorithms that are implemented using hardware techniques and bit-oriented communication protocols. The use of processing resources is, in conclusion, negligible. On the other hand, to avoid the congestion of the WSN (functions that orchestrators support nowadays) our proposal is complemented with a lightweight gateway performing simple traffic engineering operations, which do not require bidirectional communication with nodes (so global efficiency is not affected).

The rest of the paper is organized as follows: Section 2 introduces the state of the art in management and choreography solutions for WSN in tasks execution systems. Section 3 describes the study scenario and the proposed solution including all important details. Section 4 provides an experimental validation of the contribution. Finally, Section 5 and Section 6 explain some results of this experimental validation and the conclusions of our work.

## 2. State of the Art

Task execution systems are, since the beginning, one of the most important applications for WSN [11]. In these scenarios, one of the key problems is the selection of the most adequate node to execute a certain task, delegated by high-level applications. If the node, or nodes, to execute a task are selected by components in a hierarchical level superior to the nodes (such as cluster heads) it is said the network implements an orchestration management solution (see Figure 1b). On the other hand, if the nodes to execute a task are selected by means of an agreement among all nodes in the WSN (see Figure 1a) it is said the network implements a self-management solution or a choreography management solution (sometimes also named as collaborative management).

Most extended proposals about management in WSN are based on orchestration schemes [12]. In these solutions, information about node hardware capabilities is sent to the orchestrator and/or any component in charge of the network management. Then, when a task is delegated by high-level applications, a matchmaking process is performed by those components in order to find the nodes which provide the adequate functions (nodes) to execute the delegated task [13].

In general, works on orchestration schemes define a set of “node adaptors” which homogenize the control interface of nodes in order to communicate them with a middleware supported by the orchestrator [14]. This middleware is usually service-oriented and may consider artificial intelligent algorithms [14]. Some proposals include a virtual representation of the WSN in this middleware [15], so calculations and operation are performed on this virtual instance before being mapped on the real network. It is also possible to find authors describing the orchestration middleware as in mobile networks (considering a global register, a monitoring module, etc.) [16]. Finally, agent-based management architectures have been also reported. Different agent locations and manager (orchestrator) configurations were investigated [17].

As orchestration schemes need an initial configuration process (to send the information about the WSN to the manager), dynamic changes in WSN are one of the main problems in these technologies [18]. As the repetition of the initial configuration process may stop the operations in the WSN, a technique named as “context-aware management” was proposed [19]. In this technique, the orchestration middleware collects and maintains information about the entire environment and situation of nodes in the WSN. Solutions based on semantics [20] and/or pattern recognition [21] are employed to take management decision with the available information.

Any case, all previously described solutions present a common problem: they imply complex processing operations (such as analysing semantic objects, starting virtual instances, updating a huge community of agents, etc.) which make impossible to operate at real-time, except in small deployments. Thus, although managers and orchestrators have the required computational power to perform these operations, the efficiency in control transactions is very low.

In these hierarchical networks, besides, complex communication protocols and transactions are deployed and defined, as nodes in WSN must send a relevant amount of information to orchestrators. Up to ten different messages [20] may have to be exchanged by each node with the orchestrator before executing a management operation (running a delegated task, for example); and this number may grow if cluster heads or any other medium-level component is also considered [22]. These protocols and transactions, furthermore, force the nodes to maintain large state descriptions and to handle heavy data formats such as JSON (JavaScript Object Notation) [22].

In conclusion, an important part of the processing resources in nodes (which are resource constraint) is finally consumed by management operations, even when considering central orchestrators. This is especially critical when considering the processing time, as control transactions require large communication processes when various heavy messages must be created and interpreted.

These problems [23], nevertheless, may be solved if collaborative, choreographed or self-management solutions are implemented. In fact, although they are sparse, some works have investigated these techniques. Task allocation using choreographed technologies is usually understood as an optimization problem. The basic idea is to select the set of nodes which may execute a certain task or workflow consuming the minimum amount of resources. Various works have instigated the complexity of the underlying mathematical problem [24,25], proving that traditional task allocation techniques cannot be directly implemented in choreographed WSN due to the employment of resource-constraint devices [26].

Partial solutions to this problem have been reported optimizing the energy [27,28] or time [29] consumption for a given set of tasks. In order to solve the global multi-objective optimization, heuristic solutions [30] and stochastic heuristic methods have been investigated [31]. In particular different variations of the well-known Particle Swarm Optimization (PSO) algorithm may be found [32,33]. Other bionic intelligence methods have been also proposed to allocate tasks to be executed in an optimum way [34].

These techniques, however, are only valid if networks are static and the sequence of tasks to be executed is known. If real-time changes have to be considered, or the tasks to be executed are aleatory, either the optimization problem cannot be solved (furthermore, it has no sense), or the obtained solution is only a first approximation which could be obtained using much lighter processes.

In a more relaxed way, other proposals try to schedule the task execution, so most adequate nodes are in charge of it but no optimization or fitness function is defined. Geographical information or real-time operations [11] can be added as a novelty in this last group of solutions.

The main problem of all described choreographed techniques, nevertheless, is the same. The complexity and resource consumption of these algorithms grow with the number of nodes in the network [33]. Some of them, even, present an exponential evolution. Any case, software implementations of the described technologies have been reported to work only in WSN with a few tens of nodes. In current pervasive sensing platform, then, they cannot be employed, so orchestration techniques are usually preferred.

The proposed solution in this paper includes a hardware-supported algorithm which has negligible resource consumption and can be executed at real-time in large WSN, as its complexity does not depend on the number of considered nodes. No control transactions or communication processes are necessary to manage the infrastructure. The proposed choreography solution, besides, enables the self-management of nodes, so no network orchestrator has to be included and only a lightweight gateway must be considered.

## 3. Self-Managed and Choreographed Task Execution in Sensor Networks

This section describes the main contribution of this paper. First subsection presents the study scenario and the characteristics of the considered WSN. Second subsection presents a global overview of the proposed technique and the data format employed in task execution orders. The third subsection explains the behaviour of the proposed network gateway. And, finally, fourth subsection describes the proposed hardware-supported algorithm to be implemented in sensor nodes.

### 3.1. Study Scenario

Our scenario is designed for all types of environments where smart sensor nodes are deployed to perform cyber-physical operations, i.e., operations where either some information (such as temperature, humidity, presence, etc.) is gathered from the environment, or some effects (e.g., turning on the lights) are applied to the physical world. The node density is very high, because new deployments tend to be pervasive and future paradigms such as pervasive sensing platforms or smart dust present this characteristic.

Besides, hardware capabilities are redundant; so many different nodes may perform any operation within the WSN. In this initial study, we do not consider operations involving the transmission of images, video streams, sounds or other information requiring large and/or variable amount of data. Each operation consists of two messages: a bit-oriented execution order with a fixed structure and length; and a bit-oriented response including the result of the operation also with a fixed structure and length (see Section 3.2). We are also assuming the WSN performs a constant monitoring of the environment and may execute operations at any moment. However, real-time processing is not mandatory, if the application scenario does not require this characteristic (as in some agricultural systems [35]). Thus, ad hoc sensor networks and mobile nodes may be also considered. Even, other solutions, such as including the sensors in mobile nodes could be also considered if necessary.

The proposed technology belongs to the transport level in the OSI reference model [36]. As in standard Internet transactions, operation messages in WSN enable the communication of remote applications (i.e., the user application which invokes the operation and the low-level program in the smart node which finally executes it); regardless the underlying infrastructure. As in other transport level protocols, the proposed technology allows identifying the application to be invoked, considering reliable communications and generating complex transactions.

**Figure 2:**
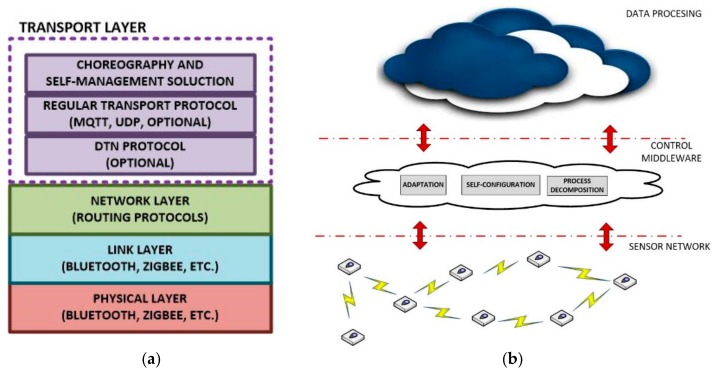
(**a**) Protocol stack in the proposed scenario (**b**) Scenario’s logical structure: layers.

As a consequence, in our scenario, all nodes must implement a network protocol proving addressing to the entire network. No special characteristics are required from this protocol, except a broadcast and/or anycast solution [37] must be supported. If needed, a Delay-Tolerant Protocol [38] could be also included (see Figure 2a).

A complete list of all admissible or preferred characteristics should be based on a deep analysis and the definition and characterization of all possible application scenarios [18] but in our case, we are going to work over some common characteristics for all cases, which are:

1. Lack of infrastructure for supporting power supply or access to unlimited data storage (such as data bases).
2. Usage of specific protocols for stablishing WSN’s topology in an autonomous way and for routing data from the user applications to the smart nodes which must perform the cyber-physical operations
3. Nodes may be mobile and stablish ad hoc connections. DTN technologies for communication among nodes and other components could be included.
4. The node density is very high and hardware capabilities are very redundant.
5. The entire WSN is deployed in a unique geographical location. Thus, cyber-physical operations considering geographic information can be executed in any available node.

The first point limits the kind of technologies we can use and more specifically, the lack of power supply limits the service time of the network, which is a key element for the usability of such kind of solutions in real environments.

The second and third points explain how smart nodes create an organized network. On the one hand, as the proposed technology belong to transport level, network level solutions must be previously considered. On the other hand, as nodes may be mobile and not be available all time, Delay-Tolerant Network (DTN) technologies to allow reliable and efficient communication could be implemented.

Finally, the fourth and fifth points are directly generated by the requirements imposed by most recent and future scenarios. Industry 4.0 systems or pervasive sensing platforms, in fact, are based on very dense networks, deployed in a unique geographical location and with a very high reliability (so several redundant nodes are included).

Three layers are identified in our application scenarios: sensor network, control middleware and data processing and user applications (Figure 2b).

Sensor network refers to a set of sensorization nodes, including transducers and processing components, being able to execute cyber-physical operations (and, in consequence, tasks, see Section 3.2). In general, sensors may be distributed in a random way (if node installation is possible in all points within the deployment area) or in a planned way (if some points are preferred). Therefore, we cannot guarantee that we know every position of the fixed nodes. In this work, we are assuming we know neither the sensors’ position nor network topology or the number of nodes. Moreover, in relation to the node characteristics, the proposed solution allows employing any type of device. That means we do not have to be worried about values such as the sensing range, the sensing period or the monitored variable; because our proposal is independent from sensors’ features and only considers generic parameters, such as the energy consumption. In that way, these assumptions make the proposal very general and flexible.

Control middleware includes all the components employed to communicate and adapt the low-level architecture of WSN and the high-level design of user applications. Process decomposition mechanisms, self-configuration instruments and many other similar solutions are included in this layer.

Data processing layer and user applications refer to all final high-level components which receive data from WSN and control middleware, process them and (eventually) store and/or present them. This layer can be based on any standard technology like centralized services or cloud services, because in our scenarios real time operations are not mandatory if the application scenario does not require them.

### 3.2. Global Overview. Protocol for Task Execution

The proposed technology is composed of two different algorithms. The first one is implemented in the control middleware, where a lightweight gateway is included in order to avoid the congestion of the WSN (guarantying the stability of the choreography solution). The second one is implemented in all nodes of the WSN, so they can self-manage their resources and the execution of the delegated tasks.

In this context, a task is understood as the sequence of various chained cyber-physical operations (CPOs), for which it must be obtained a unique and global result by the WSN. In a more formal way, a task may be represented as a workflow of cyber-physical operations. These workflows can be described using any available description language (BPEL, BPMN, etc.), although in this article we are employing YAWL [39] because of their capability to represent almost every possible situation.

**Figure 3:**
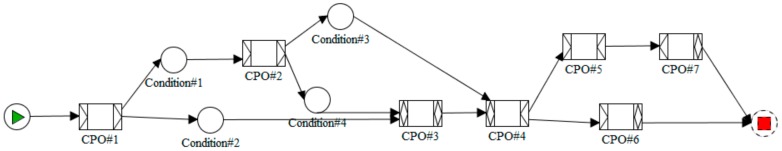
Task as a workflow of cyber-physical operations (CPO).

Delegated tasks can be received in the control middleware already described as workflows of cyber-physical operations or may be transformed in that middleware. Any case, this transformation process is not the objective this paper, so we are assuming we already have that workflow-like description (see Figure 3).

According to the Erlang’s queue theory, a lightweight gateway in the control middleware evaluates if the maximum allowed load in the sensor networks has been reached (i.e., if the network is congested). If positive, the task execution is rejected. However, in order to determine the maximum allowed load in the WSN, a statistical procedure is employed, so some rejected tasks are finally executed in order to determine if the network is in fact congested or the maximum allowed load can be increased.

Accepted tasks are transformed in a set of execution orders (one per cyber-physical operation in the task workflow), which are represented using a bit-oriented format. These orders also contain information about how they are related among them in order to guarantee that inputs and outputs of the different operations are correctly linked. This characteristic enables the use of hardware-supported technologies in nodes.

Once constructed, these orders are broadcasted in the WSN. Thus, the smart nodes in the WSN include a hardware module which, once received an execution order, decides if the node can or cannot execute this operation. In this decision, the proposed algorithm considers the available resources, the interest and importance of the operation to be executed and the predicted resource consumption made when performing the operation. These resource self-management functionalities are complemented with choreography solutions, so that nodes can decide in an autonomous way which device finally executes the operation (using pseudorandom number generators); and can also compose the final result of a delegated task from the partial results of the performed cyber-physical operations (using the identifiers and the cross-references included in the execution orders).

In this proposal, these solutions are supported using only hardware technologies, so embedded software in nodes must not be modified to include coordination algorithms and critical resources (such as the operation time) are not wasted in control operations.

**Figure 4:**
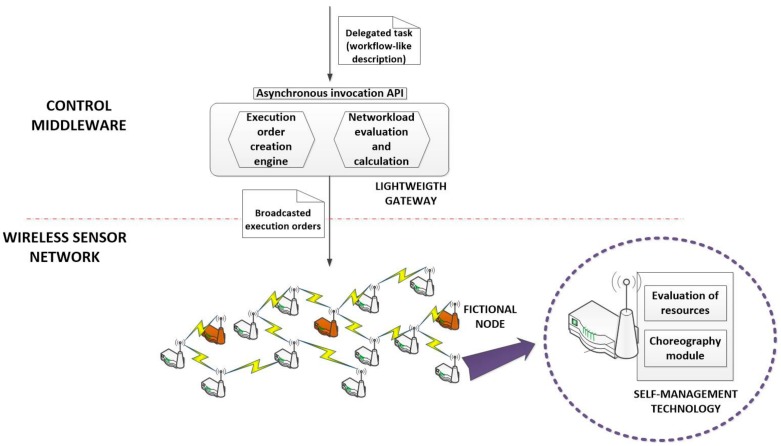
Global scheme of the proposed technology.

Figure 4 represents the global overview of the proposed technology.

Bit-oriented execution orders can be easily generated by the proposed lightweight gateway, or this functionality may be deployed in a second and new component. In this first work, we are assuming the gateway is in charge of this function (see Figure 4).

**Figure 5:**
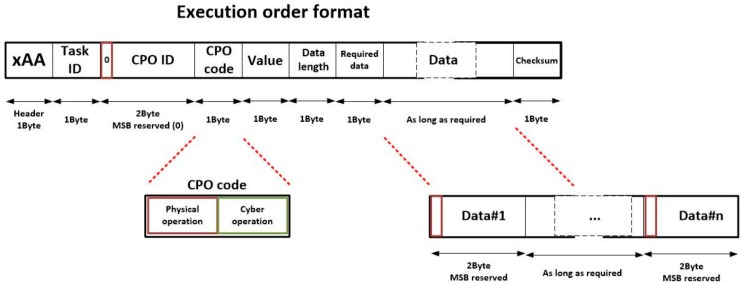
Bit-oriented execution order format.

The proposed format for the execution orders may be seen in Figure 5. This message is needed to require the sensor network to execute a CPO or, at least, to evaluate the possibilities of carrying out the execution. The proposed format is byte-aligned. Eight different fields are considered in each execution order. Namely:

- Header (1 byte). The first byte is a header, which must be completed with the hexadecimal value xAA, to indicate an execution order is described by this command.
- Task ID (1 byte). This field identifies the delegated task to which the CPO belongs. As maximum, 255 tasks can be executed at the same time.
- CPO ID (2 bytes). This field identifies each concurrent CPO univocally. Although 2 bytes are intended for this purpose, only fifteen bits are employed to represent the CPO identifier. The Most Significant Bit (MSB) is reserved and initialized with a binary zero. This bit will be used to link inputs and outputs of CPO in an easier way (see Section 3.4).
- CPO code (1 byte). This field indicated the CPO to be performed. Two hundred fifty-five (255) different operations may be considered in the WSN. In order to simplify the decision process in smart nodes about if they are able to execute an operation, the CPO is divided into two fields. The four most significant bits represent the physical operation to be performed (i.e., the transducers involved in the execution: temperature sensors, LEDs, etc.). If a node does not include that hardware no more processing is required to reject the execution. The four Least Significant Bits represent the cyber operation to be performed (i.e., obtaining an average, sending the raw collected data, etc.).
- Value (1 bytes). This field represents the value the operation has for the system. It is obtained as a composition of the importance of the delegated task for the system and the relevance of the operation within the delegated task (see Section 3.3).
- Data length (1 byte). This field indicates how many input data are required (as maximum) to execute the CPO. These data are provided in the adequate field. Each data needs 2 bytes, so the length of the data field may be calculated multiplying by two the value represented here.
- Required data (1 byte). This field indicates how many input data are required and essential to execute the CPO. This field is employed to represent the join-type of the CPO [40]. Three different join-types are possible in YAWL: AND (if an operation is not triggered until all previous operations have finished, so required data is equal to data length); XOR (if an operation is triggered once the first previous operation has finished, so required data is equal to the unit); and OR (if and operation is triggered once a certain number of previous operations -to be selected by the user- have finished, so required data includes a personalized value). Once all required data are available, the CPO is executed.
- Data (between zero and 512 bytes, as needed). In this field, all input data of the CPO are represented. Each data requires 2 bytes: the MSB is reserved (it is a flag) and the next 15 bits includes the value of the datum. If the datum is a constant, the MSB is initialized with a binary one and the next 15 bits include the value of the constant. However, on the other hand, if the datum is obtained as the output of a previous CPO, the MSB is initialized with a binary zero and the next 15 bits include the CPO ID of the operation which will produce the datum. The order in which data are listed must be fixed and established for each operation.
- Checksum (1 byte). The checksum is obtained as the XOR of all bytes in the execution order except the header. If an order is corrupted, the checksum will not be validated and the order will be discarded.

**Figure 6:**
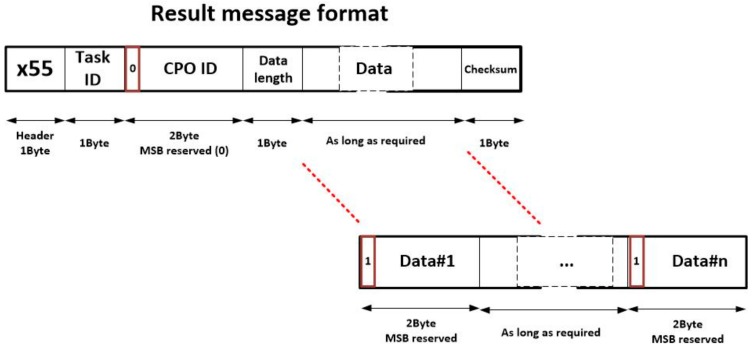
Bit-oriented result message format.

Once a CPO is performed, the corresponding node generates a message including the results of the operation, in order to allow the other nodes to execute the linked operations. This message is needed to update the state of the choreographed task execution. The format of this message is showed on Figure 6. As can be seen, fields in this new message are similar to which previously presented. In particular, six fields are considered:

- Header (1 byte). The proposed header for result messages is x55
- Task ID (1 byte). This identifier has the same meaning as explained above.
- CPO ID (1 byte). This identifier has the same meaning as explained above.
- Data length (1 byte). This field indicates how many output data are generated as result of the CPO. These data are provided in the adequate field. Each data needs 2 bytes, so the length of the data field may be calculated multiplying by two the value represented here.
- Data (between zero and 512 bytes, as needed). In this field, all output data of the CPO are represented. Each data requires 2 bytes: the MSB is reserved (and initialized with a binary one) and the next 15 bits includes the particular value of the result. The order in which results are listed must be fixed and stablished for each operation.
- Checksum (1 byte). The checksum is obtained as the XOR of all bytes in the result message except the header.

As can be seen in Figure 3, sometimes workflows may include conditions, in order to select which branch (or branches) in the workflow is executed. These structures can appear depending on the split-type of operations. In YAWL, three different split-types are defined: AND, OR and XOR (with the same meaning that the join-types). Tasks of OR or XOR split-types must include conditions [40]. Conditions must be also executed, so they are treated as any other CPO (they have a CPO ID, they present cross-references with other operation in the data field, etc.). However, as the code to execute these conditions is not available in the nodes of the WSN (as conditions are defined at high-level by users considering the application scenarios and not the underlying network), they are not executed by hardware nodes. Execution orders of conditions are not neither broadcasted and all of them are resolved by fictional nodes (see Figure 4). Fictional nodes are virtual instances hosted in the control middleware but they behave and relate with other nodes as any other standard hardware component. In these nodes, the lightweight gateway may deploy dynamically the required code to execute the conditions in the workflow.

**Figure 7:**
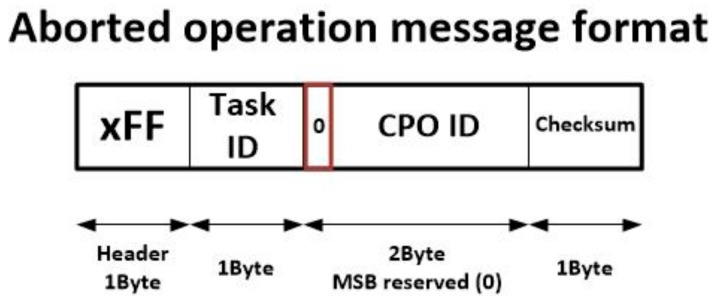
Bit-oriented aborted operation message format.

Some conditions will be verified, so a standard result message will be generated. However, other conditions could not be verified and nodes in charge of executing the operations in the cancelled branch (or branches) should deallocate the corresponding resources and be available for new operations. In order to enable that, a new message, the aborted operation message, was included in the proposed task execution protocol (see Figure 7). This message is much shorter and fields have the same meaning as previously described commands.

**Figure 8:**
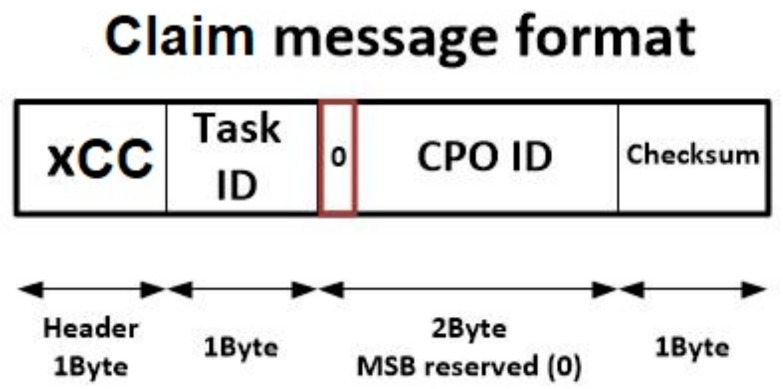
Claim message format.

Finally, although at this point the necessity of including this message may not be seen clearly, it a message is needed to enable nodes to claim the right of executing a CPO ordered by the gateway. This last message is named “claim message” and its structure is very similar to the aborted message format, although the header, in this case, is initiated with the value xCC (see Figure 8). This message is totally necessary, as it is employed to ensure that (in a choreographed execution algorithm) only one node is in charge of executing each CPO. Among nodes willing to execute a CPO, a random mechanism is employed to select the final node responsible of the execution. This node must claim that execution, so the other nodes can renounce it.

Considering the proposed messages, smart nodes in the WSN may execute tasks in a choreographic way, as described in Section 3.4.

### 3.3. Network Gateway

The main problem of choreography solutions is that no node in the sensor network has a global understanding of the ongoing situation. In that way, networks implementing these technologies may get congested easily, as no node can reject the execution of a task or operation on behalf of the entire network. Timers and other similar solutions must be considered in this case in order to inform high-level applications that a task cannot be executed. However, those techniques do not enable the premature detection of congestion situations, which makes them very inefficient.

Therefore, in the proposed solution, a lightweight gateway (see Figure 4) has been included. This gateway does not need to maintain a real-time representation of the network state and only using statistical procedures can detect and prevent the congestion of the network.

A Wireless Sensor Network W may be seen as the union of a family of sets of servers (1). Each set of servers $S_{i}$ groups the nodes being able to perform a certain CPO. As, usually, a node may execute various different operations, it may be present in several server sets.

(1) $$W=\overset{m}{\underset{i=1}{\cup }}S_{i}$$

The cardinality of each set of servers (2) is usually unknown, as configuration processes are not able to collect that information in most cases. The total number of nodes in the WSN is $N_{total}$.

(2) $$N_{i}=card\left\{S_{i}\right\}$$

In this context, delegated tasks are received by the WSN in a random manner. Nevertheless, it is reasonable to imagine that the probability distribution of task delegations (and execution of CPO) in time in WSN $f_{d}\left[k\right]$ is similar to the probability distribution of calls in time in telephony systems (3). In particular, delegations are independent among them and they are equiprobable in time; so a Poisson distribution is the most adequate.

(3) $$f_{d}\left[k\right]=\frac{{\left(\lambda_{i}T\right)}^{k}}{k!}e^{-\lambda_{i}T}$$

**Figure 9:**
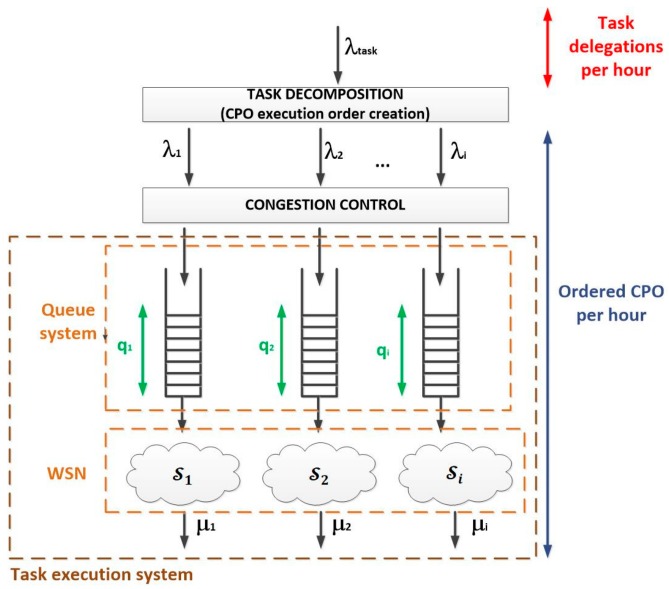
Queue system in the proposed solution.

Then, for each server group $S_{i}$, the probability of receiving k task delegations in a time period T is represented by a Poisson distribution with medium $\lambda_{i}T$; where $\lambda_{i}$ is the average rate of received delegations (or ordered CPO, depending on the point of view, see Figure 9) by the group of servers $S_{i}$ during the time period T.

Experimentally, it is proved that the service time follows an exponential probability distribution in telephony systems. Although no similar results have been obtained for WSN, in this work we are assuming the service time in WSN follows an exponential law as well (4). Hereinafter, $\frac{1}{\mu_{i}}$ is the medium service time for the group of servers $S_{i}$ (i.e., the required time to execute a certain CPO operation by a server within $S_{i}$).

(4) $$f_{st}\left(t\right)=\mu_{i}e^{-\mu_{i}t}$$

As, in some occasions, bursts of task delegations may appear, for each group of servers it is considered a finite queue $Q_{i}$ to absorb the effect of these bursts. Each queue $Q_{i}$ has a maximum capacity of $q_{i}$ CPO execution orders. The resulting system is a hybrid queue system whose Quality-of-Service (QoS) is determined by both the waiting time in the queue and the loss probability due to the limited capacity of the queues.

Although this model considers to be defined some assumptions that are met in most cases, the traffic engineering theory guarantees that the obtained results are a good first estimation for all cases, including those which do not fulfil the initial assumptions (for example, if task delegations are periodic). General models as the proposed one by Kingman [41] could be applied for a more precise calculation.

In this scenario, it is possible to calculate the volume of CPOs $A_{i}$ supported by each group of servers $S_{i}$ using the Erlang’s theory (5). It is also possible to calculate the congestion factor (or utilization factor) $\rho_{i}$ which represents (if $\rho_{i}>1$) a permanent and structural situation of congestion in the WSN (6)

(5) $$A_{i}=\frac{\lambda_{i}}{\mu_{i}}$$

(6) $$\rho_{i}=\frac{A_{i}}{N_{i}}=\frac{\lambda_{i}}{N_{i}\;\mu_{i}}\;\;$$

With this approach the situation of the WSN, regardless how great is the node density or the number of nodes, may be represented using only five one-dimensional arrays (with 256 values each one, one value per CPO). These arrays represent the medium task delegation rate, the inverse of the medium service time (or performed-served-operation rate), the number of servers and the congestion factor (and/or the traffic volume) in the WSN (7).

(7) $$\vec{N}=\left\{N_{i}\;,\;\;\;i=0,\ldots ,255\right\}\;\vec{A}=\left\{A_{i}\;,\;\;\;i=0,\ldots ,255\right\}\;\vec{\lambda }=\left\{\lambda_{i}\;,\;\;\;i=0,\ldots ,255\right\}\;\vec{\mu }=\left\{\mu_{i}\;,\;\;\;i=0,\ldots ,255\right\}\;\vec{\rho }=\left\{\rho_{i}\;,\;\;\;i=0,\ldots ,255\right\}$$

As in standard telephony systems, in this case we are assuming the number of users that try to use the resources of the WSN is very high. However, users have a variable behaviour in time, i.e., the situation of the WSN and the values of the mentioned arrays (7), will vary in time (although this dependency is not explicitly expressed). Because of this variability, in order to dimension systems, key indicators are evaluated during the most loaded hour (such hour when the medium task delegation rate is highest). An important topic in every WSN for task execution, then, will be to guarantee that, at no time, the hourly ordered CPO rate is above the calculated rate for the most loaded hour $\vec{\lambda_{max}}$. The use of these “maximum” values as comparison threshold it will be very important to maintain the network in a stable situation as we are seeing.

Two relevant considerations have to be done at this moment. First, the module of vectors $\vec{A}$, $\vec{\rho }$, etc. may be used as a first evaluation of the situation of the network as a whole. However, the obtained result it is not precise from the traffic engineering point of view. And, second, among the presented arrays (7) only two of them are not modifiable as they depend on the deployed hardware devices: the number of servers $\vec{N}$ and the inverse of the service time $\vec{\mu }$. The other three parameters are dependent on each other, there is only one degree of freedom, i.e., only one parameter can be employed as independent variable. In this work, we employ $\vec{\rho }$ as such an independent variable, which is the basis of the congestion control algorithm to be deployed in the lightweight gateway (see Algorithm 1).

According to the Erlang’s theory, the loss probability any of the previously described queue systems, for each group of servers, is (8). In this work, we are assuming the system must present a certain QoS level, represented by the availability of the system (expressed as probability) for each group of servers, $p_{av}^{i}$. Then, the maximum allowed loss probability in the system $p_{loss}^{i}$ is (9). As in the previous case, one-dimensional arrays of 256 elements may be employed to represent the loss probability and availability of the entire network $\vec{p_{loss}}$, $\vec{p_{av}}$.

(8) $$p_{loss}^{i}=\;\rho^{q_{i}+1}\frac{1-\rho }{1-\rho^{q_{i}+2}}$$

(9) $$p_{loss}^{i}=1-\;p_{av}^{i}$$

**Figure 10:**
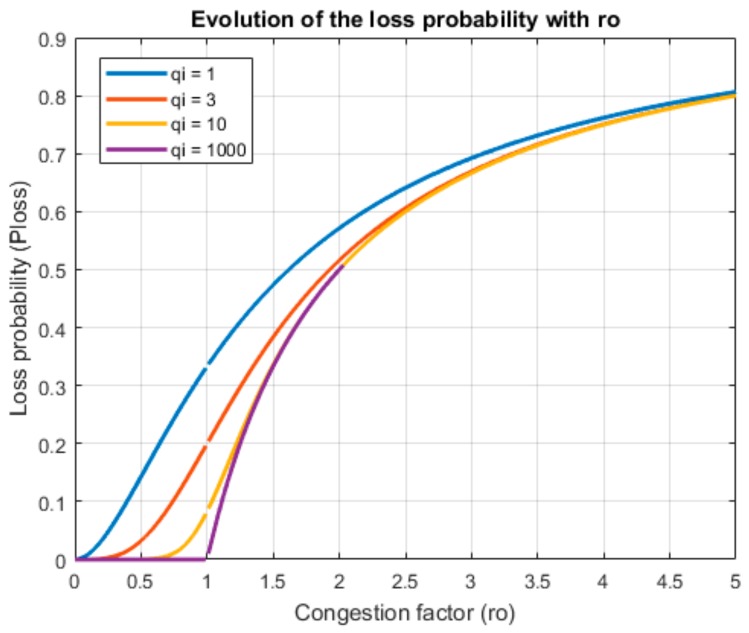
Evolution of the loss probability with the congestion factor.

With these equations, it is possible to determine the maximum utilization factor and maximum hourly task delegation rate which can support the WSN, to guarantee the desired QoS. There is no analytic solution but it is pretty easy to obtain a numerical one (see Figure 10).

Using a statistical procedure and a sliding window filter (we have named the “Erlang filter”) only task delegations compatible with the desired QoS will be accepted in the system (see Algorithm 1).

As can be seen (Algorithm 1), the first activity to be performed when a task delegation is received is evaluating the number of CPO of each type which will be executed if the task is accepted. This step is very important, as tasks are delegated as a unique element but they are decomposed into CPO in the WSN. Congestion factors and the other traffic engineering parameters are, then, expressed terms of the ordered CPOs, so it is basic to know how many CPO will be executed per delegated task.
**Algorithm 1** Lightweight gateway (congestion control algorithm)**Input:** Delegated task $t_{del}$
Calculate set_cpo, the set of CPO composing $t_{del}$Create an array number_cpo[256]Calculate the current time in Epoch format date**for each** CPO cpo in set_cpo
**do**   Add one unit to Number_cpo[(int)cpo]
**end for**Create a Boolean variable task_accepted equal to ‘true’**for each** element number in number_cpo
**do**  Calculated the number past_task of ordered CPO between date and date – 1h by $S_{i}$
  
**if**
$\;past\_task+number\;>\;\lambda_{max}^{i}$
**then**   task_accepted is false  **end if****end for****if** task_accepted is true **then**  CPO in set_cpo are introduced in the task execution system
**end if**


As CPO are identified by one-byte integer numbers, a simple casting of data types enables us to count the amount of CPO of each type that make up the delegated task.

**Figure 11:**
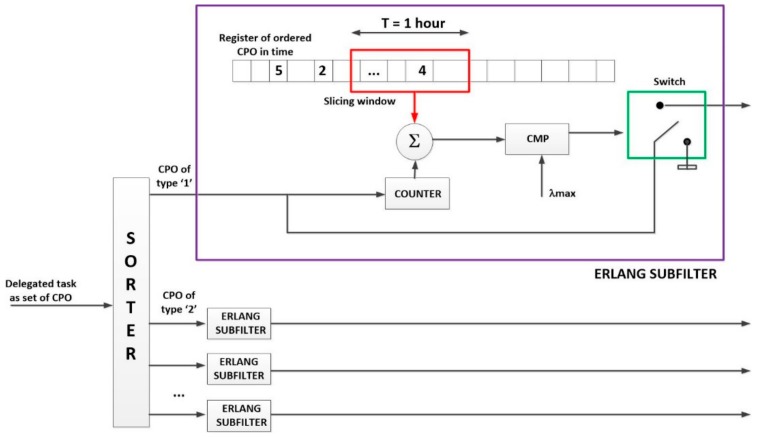
Structure of the proposed Erlang filter.

The rest of the Algorithm 1 is the proposed “Erlang filter,” designed to avoid the congestion in the system. Even if queues in the devices and/or the gateway could store more CPO than admitted by this filter (for example, if queues were infinite), the system becomes unstable if the utilization factor is higher than one, so this filter prevents the queue system to reach that point (see Figure 11).

The basic idea is to store a historical register of the amount of ordered CPO (not received tasks, as rejected tasks must not be included) in time. A slicing window with a width of T time units (usually an hour to be coherent with the traffic engineering principled) moves through the registry and accumulates all the operations that fall within it. The value of T parameter should be chosen to be large enough to remove the random fast variation in the task delegation flow; but short enough to allow observing the slow variations that occur throughout the day. Although specific values for particular applications could be calculated, the traffic engineering theory has proved that, in the general case, one hour is a good balance between both requirements. If the accumulated value (considering also the number of operations which will be introduced if the task is accepted), for any server group, is higher than the maximum allowed hourly medium CPO ordination rate, the delegated task is directly rejected, no operation is introduced in the queues.

Thus, a key problem is to calculate the maximum allowed hourly medium CPO ordination rate $\lambda_{max}^{i}$ for each group of servers. Considering that, as said, the maximum value of the congestion factor is obtained to fulfil the requirements of the desired QoS; and $N_{i}$ and $\mu_{i}$ are imposed by the hardware characteristics of the WSN, this maximum rate is easily calculated (10).

(10) $$\lambda_{max}^{i}=\;\rho_{i}\;\cdot N_{i}\;\cdot \mu_{i}$$

Then, it is necessary to calculate the values of $N_{i}$ and $\mu_{i}$ of the underlaying WSN. The calculation of $\mu_{i}$ is simpler and it is showed in Algorithm 2. On the other hand, Algorithms 3 and 4 are designed to estimate the value of $N_{i}$, which is more complicated as this parameter cannot be directecly measured.
**Algorithm 2** WSN parameter calculation. Service time**Input:** Circular matrix of service time measures serv_measure
   Array number_cpo[256] of CPO to be executed**Output:** Medium service time $\vec{\mu }$
Obtain the current date in Epoch format, create a variable initial_date
Create an integer pending_op equal to one**while** pending_op is higher than zero **then**  pending_op is equal to the addition of elements in number_cpo
  Wait for the result of an operation  Subtract one unit to number_cpo[(int)result.CPO_ID]  **if** received result is not a cancellation **then**   Obtain the current date current_date in Epoch format   Store the new measure current_date – initial_date in serv_measure in the row (int)result.CPO_ID  **end if****end while****for each** server group $S_{i}$
**do**   Integer num_measure is equal to number of non-zero elements in i-th row of serv_measure
   Acc_time is equal to the addition of all non-zero elements in i-th row of serv_measure
   
$\mu_{i}=\;Acc\_time\;/num\_measure$

**end for**

**Algorithm 3** WSN parameter calculation (1)**Input:** Delegated task $t_{del}$  Previous estimation of $\vec{N}$ (if exists)**Output:** Estimated $\vec{N}$**if** it does not exists **then** create a Boolean variable init_time equal to True**if** init_time is true **then**  Calculate set_cpo, the set of CPO composing $t_{del}$
  Create an array number_cpo[256]
  **for each** CPO cpo in set_cpo
**do**   Add one unit to Number_cpo[(int)cpo]
  **end for**
  CPO in set_cpo are introduced in the task execution system
**else**
  Algorithm 1**end if**Create a Boolean variable empty_queue equal to ‘false’**if** all queues $Q_{i}$ in the task execution system are empty **then**  empty_queue is equal to True
**end if**
**if** execution orders have been sent to the execution system **then**  Algorithm 2  Configure a timer  Wait for the result of the delegated task  **if** timeout and result is not received **then**   Send a cancellation message for all CPO   **if**
init_time is True **then**    init_time is false   
**else**
    All elements in $\vec{N}$ are divided by two   
**end if**
  **else**
   **if** empty_queue is equal to True **then**     Algorithm 4   **end if**
  **else**
   Generate a random number num
   **if**
num is higher than threshold T **then**    CPO in set_cpo are introduced in the task execution system    Configure a timer    Wait for the result of the delegated task    **if** timeout and result is not received **then**     Send a cancellation message for all CPO     Return    **else**
     **if** empty_queue is equal to True **then**
      Algorithm 4     **end if**
   **end if**
    Update threshold T

**end if**

**Algorithm 4** WSN parameter calculation (2)**Input:** Array number_cpo[256] of executed CPO    Array pending_cpo[256] of CPO being executed    Previous estimation of $\vec{N_{old}}$ (if exists)    Thresholds p_high and p_low
**Output:** Estimated $\vec{N}$Integer discover_nodes is the addition of elements in number_cpo and pending_cpo
Integer current_nodes is the addition of elements in $N_{old}$
**if** number_cpo is higher than $N_{old}$
**then**  Calculates p the p-value using the Mann-Whitney *U* test  **if** p is smaller than p_high and higher than p_low
**then**    $\vec{N}$ is obtained as the average of $\vec{N_{old}}$ and number_cpo[]+pending_cpo[]  **else if**
p is smaller than p_high and smaller than p_low
**then**    $\vec{N}$ is equal to number_cpo[]+pending_cpo[]
  **end if**

**end if**


Because of the need of listening all interactions among nodes in WSN to estimate the medium service time, Algorithm 2 should be hosted in fictional nodes (see Figure 4), which are virtual entities maintained and supported by the gateway. The proposed Algorithm 3 considers a circular matrix where measures about the service time are stored (columns represent different time instants and rows different server groups). The number of elements per row k represents the number of measures employed to calculate the medium service time.

Algorithm 2 receives information about the amount of CPO of each type to be performed. Then, it waits until all of them have been executed, or cancelled (in this case the new calculated medium service time will be equal to the previous one because of the algorithm design). Considering the initial time when the delegated task was triggered and the finishing time of each CPO, a new measure of the service time is obtained (and stored in the circular matrix). It is important to remark that, as we are seeing in Section 3.4, resources of nodes get “allocated” once CPO are broadcasted and accepted. Thus, although an important time of the service time may be employed in waiting until the previous CPO in the task workflow are finished, it must be considered the whole time since CPO executions are ordered.

Once all CPO have been resolved, executed or cancelled, the medium service time is evaluated considering the new data and all previous k−1 samples. It is important to note that, if we assume the WSN is not congested, the service time may be considered independent from the hourly task delegation rate (and only dependent on intrinsic hardware and software factors). Thus, all measures can be employed to calculate the average, regardless the moment and external conditions when they were acquired.

Moreover, as these kind of “learning algorithms” require a certain time to converge to a stable value (while enough data are accumulated), only non-zero values in the buffer of measures will be considered to obtain the medium service time.

**Figure 12:**
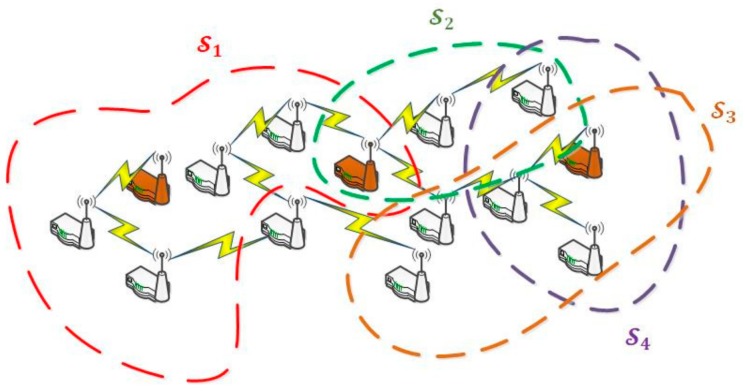
Distribution of nodes in the different server groups.

Algorithm 3 is the most complex algorithm to be described in this work. It makes reference to all previously described algorithms, as the evaluation of the number of nodes of each type in the WSN is a key problem without a simple solution. As we said, nodes are considered servers in our model, grouping them according to the CPO they can perform. As, usually, each node can perform various CPO, the same node might be included in various server groups (see Figure 12). Nevertheless, at each time instant, a hardware smart node may only act as belonging to one group $S_{i}$ (i.e., it can only perform a CPO at the same time, as sensor nodes usually do not allow parallel programming), although this association can change over time.

When estimating the number of servers per group, $N_{i}$, however, it is desirable the values to be stable over time. Oscillating variables cause the algorithms to behave in a random way and we could no guarantee the WSN is not congested. Although different policies may be employed to fix stable values for $N_{i}$ (e.g., distributing the nodes homogeneously among the shared groups), in this case these values are adjusted to the real use demand of the different server groups. Then, server groups receiving more CPO execution orders will include more shared nodes than less used groups.

The manner in which Algorithm 3 does this data inference is by monitoring the real capacity of WSN to execute the delegated tasks. During the initialization period, most common operations will need more nodes to be executed and Algorithm 3 will include these nodes in the adequate group, although they could execute other different CPO.

Algorithm 3 considers a first initialization period, when no control about the WSN congestion is performed. Information about the underlying hardware is collected during this time, which finishes once the WSN is not able to execute a delegated task (i.e., when a previously configured timer expires and no result is received). At this moment, the number of nodes in each server group $N_{i}$ is fixed to be equal to the last value for which tasks were successfully executed.

If a congestion situation is detected (because a delegated task is not executed when the maximum allowed rate has not been exceeded) and the initial time is over, the current number of nodes in each server group $N_{i}$ is divided by two. From this point, the number of nodes in each server group should be updated until reaching its real values. This policy, in fact, must be proved to be successful in other congestion control solutions such as TCP.

Using only the previously described technique, the number of nodes will be always fixed to the value calculated during the initialization period. In order to update this value, for example if new devices are added or during the recalculation process after a congestion situation, tasks marked to be rejected can be finally processed. If these tasks are not executed by the WSN, the number of nodes is not updated. On the contrary, if these tasks are in fact executed by the WSN, that may mean more nodes than the system knows are available and vector $\vec{N}$ is updated according to this new information.

In order to select which tasks marked to be rejected are finally processed, a statistical process is developed: a random number is generated, if the number is higher than a threshold, the task is tried to be executed. The key problem in this solution is, then, the calculation of the threshold T. This threshold has to be smart and have memory, as depending on the result of previous discovering attempts it should be more difficult or easier to do a new attempt. Considering these ideas, the proposed function (11) to calculate the threshold T includes two branches, depending on if the previous discovering attempt was successful (s=1) or not (s=0).

(11) $$T\left[n_{0}\right]=\{\begin{array}{c} \frac{P}{2}\left(1+T\left[n_{-1}\right]\;\right)\;\;\;\;\;\;\;if\;s=1\\ \frac{P}{2}T\left[n_{-1}\right]\;\;\;\;\;\;\;\;\;\;\;\;\;\;\;\;\;\;\;if\;s=0 \end{array}$$

The proposed function is based on the geometric series, it is recursive and evolves between zero and P, where P is maximum number the random number generator in Algorithm 3 may deliver. In order to maintain the total randomness at the beginning, the initial value of this threshold is designed to be $T\left[0\right]=\frac{P}{2}$. The proposed function is inspired on the traditional congestion control mechanisms, that may be found (for example) in most implementations of the TCP protocol. These solutions consider exponential-like functions (like the proposed one) as they allow making the most of network resources and detect congestion situations more quickly.

The last detail to be discussed is about the updating algorithm of array $\vec{N}$. If all queues in the task execution system are empty, it is guaranteed that all pending and ordered CPO are been executed simultaneously. Then, the number of nodes in each server group must be, at least, equal to the number of CPO being executed at the same time.

If the number of discovered nodes is higher than the previously known amount, then, the vector $\vec{N}$ should be updated considering the new information. However, as each node can perform several different CPO, small differences may appear but not due to the discovery of new nodes but because of movements of nodes among different server groups. Therefore, before updating the vector $\vec{N}$ it must be guarnteed that the new array presents a globally and significant improvement in the number of discovered nodes in respect to the previous values. In order to do that, Algorithm 4 considers a statistical test: the Mann-Whitney *U* test. The Mann-Whitney *U* test is a nonparametric test of the null hypothesis that two samples come from the same population against an alternative hypothesis, comparing the mean values of the two samples. It is used to evaluate if two different data populations are similar or different (higher or lower). The p-value indicates the significance level of Mann-Whitney *U* test.

Using this test, only when a significantly better array is calculated, the values in $\vec{N}$ are updated. In practice, two different thresholds for the p-value are considered. The first one determines if array $\vec{N}$ must be updated or not. The second inicates if the actualization must be total (the new vector replaces the old one) or partial (both arrays are combined, in this case calculating the average).

### 3.4. Choreography Algorithm

Using the previously described smart algorithms, we guarantee the WSN remains in a stable state and choreography algorithms are, then, guaranteed to be effective. Besides, as the state of the WSN may be represented using a data structure with a fixed memory consumption, independent from the number of nodes in the WSN, the gateway may be lightweight and operate at real-time. Considering these previous results, a hardware-supported algorithm is a valid solution to enable smart nodes in WSN to self-manage their resources and execute tasks in a choreographed way.

**Figure 13:**
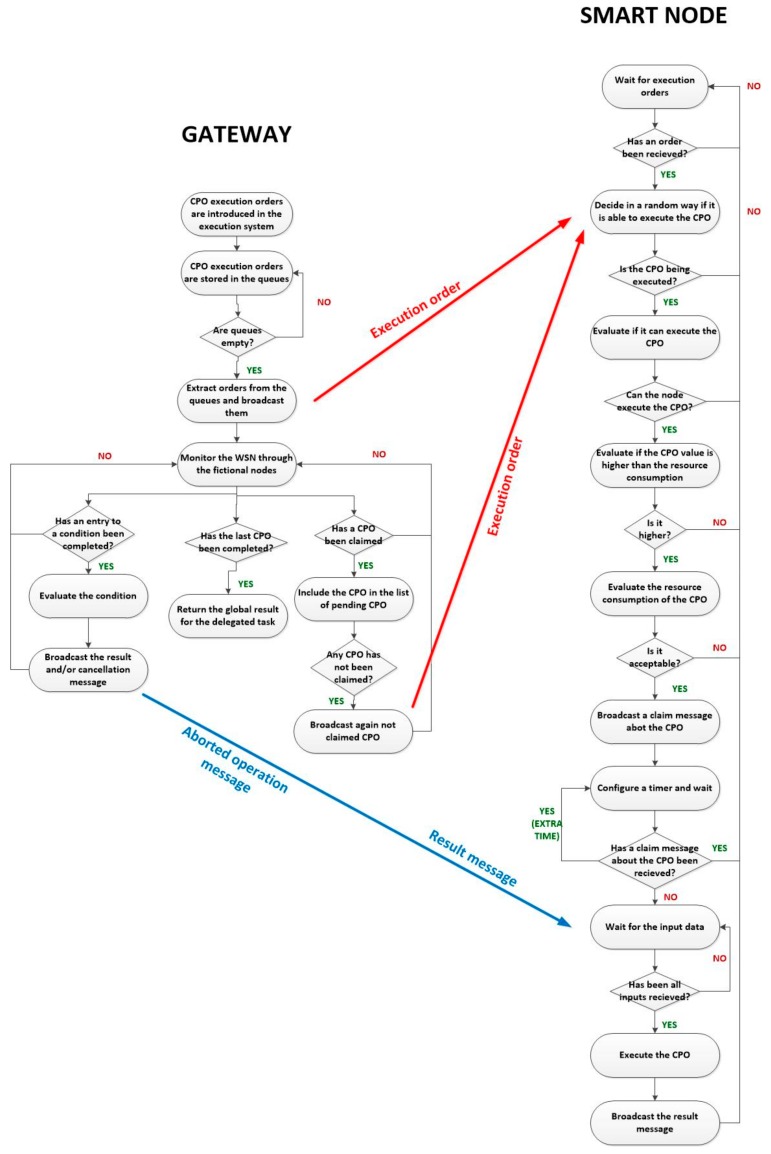
Workflow of the proposed smart task execution algorithm.

Figure 13 shows the workflow of the proposed smart self-management and choreographed task execution algorithm for WSN. The algorithm starts when the gateway introduces the corresponding CPO execution orders of a delegated task in the execution system. When nodes get available, those orders are extracted from the queues and broadcasted within all the WSN (using the message described in Section 3.2). When nodes listen these execution orders, each one decides randomly, CPO by CPO, if they would be able to execute that operation. This decision can be made using a random number generator and a fixed threshold (if the generated random number is above the threshold the CPO is admitted). After a certain time and as node density is very high, all CPO will be tentatively accepted by several different smart nodes. At this moment, before doing any additional processing, the node verifies if it can perform the required CPO. If the ordered CPO is not among the operations the node may solve, all resources are deallocated and the node returns to the initial value. On the contrary, the self-management functions are activated.

Then, if CPO is tentatively accepted, each node must evaluate two conditions in an autonomous manner. First it must be guaranteed the value of the CPO to be performed is higher than the current value of the “gas” remaining in the node. In this context, the notion of “gas” may be understood as a homogeneous variable, obtained from the aggregation of remaining resources. As the remaining amount of “gas” goes down, its value increases. Only operations whose value is higher than the value of the “gas” they are going to consume are executed. Second, apart from the value of the “gas”, the node must evaluate if it has enough resources to execute the CPO. For example, although the value of the CPO was the highest, the operation could not be executed if it requires more than a certain time (because the node was very demanded), it would run out of battery, etc.

These two decisions represent the self-management capacity of smart nodes, as they can manage their resources in an autonomous way. Later, both processes will be explained with details.

If both previous conditions are verified, the CPO is finally accepted by the node. When an operation is accepted, the node runs a timer initialized with a random time. At timeout, the node will generate and broadcast a claim message about the accepted operation. However, if before timeout, the node receives a claim message from another node (which configured the timer with a lower value), all resources are released and CPO is not executed.

Two considerations have to be done. First, if (because of the randomness of the process) a CPO is not accepted by any node, the gateway, when no node claims the execution, will broadcast the execution order again. The entire task execution is aborted if a CPO cannot be executed after various attempts. Second, as messages need a time to be transmitted, at timeout (after broadcasting the claim message), an extra time for listening for claim messages from other nodes is considered. Claim messages received during this extra time are considered to collide. In order to resolve this situation, a new random timer is configured and the process is repeated but only by the nodes that collided.

The proposed mechanism to decide if a CPO is finally executed may seem complicated but once implemented using hardware technologies, the entire algorithm will be executed almost instantly.

After all this process, only one node will be in charge of executed each CPO in the delegated task. CPO associated to conditions in the workflow of the delegated task are directly assigned to fictional nodes, through which the gateway also monitors the behaviour of the hardware devices in the WSN.

CPO whose input data are only constants (the corresponding flag in those fields is fixed to a binary one) are directly executed. When a CPO is executed, the corresponding node broadcast a result message, describing its output. In that way, all nodes in charge of CPO whose input data refer to this operation will be able to replace this reference by the final constant value to be employed (the obtained result). Once the number of “required data” by any CPO is reached (because all the needed cross-references with other CPO have been solved) the CPO is directly executed and its result broadcasted.

The gateway, once received the result for the last CPO in the workflow in the delegated task, returns the global result of the execution (equal to the result of the last CPO) to the high-level applications.

The proposed algorithm solves the choreographed execution of tasks in the WSN, however, self-management functions require a more detailed discussion.

The idea of “gas” is a global representation of the available resources in a node. The initial amount of gas in a node $g_{0}$ is a parameter of the proposed algorithm. In order to prioritize the most important CPO at each moment, as the available resources go down, the gas is scarcer and then more valuable. Only CPO whose value $v_{cpo}$ is higher than the value of the gas $v_{g}$ it is going to consume are executed. In the proposed solution, the amount of gas each low-level instruction consumes in each node is fixed, so the amount of gas required to execute each CPO is also known and fixed. If the CPO is executed, the amount of consumed gas is subtracted from the remaining gas in the node. The problem, then, is evaluate the value of this gas.

As in standard economic products, the value of each unit of gas follows an exponential evolution. As gas is scarcer, its value increases in an exponential way. In order to make comparable the CPO value and the gas value, both variables take values within the interval [0, 255] (12). Several different works have proved that (in standard economy), as the available quantity of a product decreases, its price rises exponentially [42,43]. Since the resources of the sensor network are consumed, ultimately, by the users who order the task delegations, it is reasonable the cost to grow in the same way that the price of any other resource, as it gets scarcer.

(12) $$v_{g}=\lceil \left(v_{g}^{max}-v_{0}\right)\left(1-e^{-\left(\frac{k_{inf}}{g_{t}}\right)}\right)+v_{0}\rceil$$

In the proposed assessment function, $g_{t}$ represents the amount of remaining gas at the current moment. $v_{0}$ reprsents the desired initial value for each unit of gas, as a minimum value $v_{0}=0$. $v_{g}^{max}$ represents the desired final value for each unit of gas, as a maximum value $v_{g}^{max}=255$. Finally, $k_{inf}$ is the inflaction constant. It indicated how fast the value of the gas increases. Approximately, for $g_{t}=\;\frac{k_{inf}}{5}$ the gas reaches its maximum value.

Although this expression may seem complex, in fact only one variable is present, $g_{t}$. The other parameters are design constants with a fixed value. The idea of “gas” can be employed, besides, to implement enhanced QoS techniques, even though in this paper we are not considering this option.

The implementation of exponential functions using logical gates and similar hardware solutions it is very complicated. Therefore, in order to only employ binary adders and multipliers in the proposed hardware-supported algorithm (thus, the solution can be implemented using microelectronic techniques), the Taylor’s series of the proposed function is calculated and employed (13).

(13) $$v_{g}=\;\lceil \overset{\infty }{\underset{j=0}{\sum }}\frac{v_{g}^{\left(j\right)}\left(g_{0}\right)}{j!}{\left(g_{t}-g_{0}\right)}^{j}\;\rceil \approx \;\lceil v_{0}+\left(v_{g}^{max}-v_{0}\right)\frac{k_{inf}}{g_{0}{}^{2}}\left(g_{0}-g_{t}\right)\rceil$$

Regardless the value of gas, smart nodes should consider other limits: they should never spend all their resources; they should be operating during, at least, a minimum time to be profitable, etc. In order to fulfil these requirements, nodes should be able to predict the future state of their resources at each moment.

The state of the resources in a node may be represented through a column vector $\vec{r_{i}}$ (14), where M is the number of resources to be considered. The resources of the entire WSN can be represented, then, by means of a matrix $\vec{r}$ where each column represents a different node (15).

(14) $$\vec{r_{i}}=\;\left[\begin{array}{c} r_{i}^{1}\\ r_{i}^{2}\\ \begin{array}{c} \ldots \\ r_{i}^{M} \end{array} \end{array}\right]$$

(15) $$\vec{r}=\left[\begin{array}{ccc} r_{i}^{1} & \ldots & r_{N_{\mathrm{total}}}^{1}\\ \ldots & \ldots & \ldots \\ r_{i}^{M} & \ldots & r_{N_{\mathrm{total}}}^{M} \end{array}\right]$$

In this context, the future state of the resources in the WSN, at any moment, could be evaluated through a dynamic system $\vec{F}$ depending on the current state of the resources and a stochastic process representing the task delegations. This stochastic process Φ, as said, follows a Poisson’s distribution (16).

(16) $$\dot{\vec{r}\left(t\right)}=\vec{F}\left(\vec{r}\left(t\right),\;\Phi \right)$$

If we particularize the model in a unique node, parameters in the dynamics might be divided into three elements (17): a column vector representing the resource self-management in the node (the future state of resources depends on the self- management policies applied by the node); a matrix ${\vec{r}}_{cho}\left(t\right)$, named choreography term, representing the resource state in the rest of the WSN (because of the choreography algorithm, the resource consumption in nodes depends on the behavior of the other devices); and the previously described stochastic process Φ.

(17) $$\dot{\vec{r_{i}}\left(t\right)}=\vec{F}\left({\vec{r}}_{i}\left(t\right),{\vec{r}}_{cho}\left(t\right)\;,\;\Phi \right)$$

From the point of view of a particular node, the impact of the choreography term in the consumption of its resources is a random contribution, as it does not have information about the management the other nodes do with their resources (18). This random contribution can be understood as a stochastic process $\Phi_{cho}$ representing all CPO the other nodes have not accepted. As nodes in the WSN operate in an independent way and there are a high number of them, $\Phi_{cho}$ may be assumed to be a Possion distribution (18). Stochastic terms may be, finally, grouped (19).

(18) $$\dot{\vec{r_{i}}\left(t\right)}=\vec{F}\left({\vec{r}}_{i}\left(t\right),\Phi_{cho}\;,\;\Phi \right)$$

(19) $$\dot{\vec{r_{i}}\left(t\right)}=\vec{F}\left({\vec{r}}_{i}\left(t\right),\Phi_{total}\right)$$

At this point, the proposed model using differential equations and continuous time must be transformed to a model with finite differences and discrete time (20). Besides, if we assume that all resources are independent (the memory consumption is independent from the battery discharge, for example), the dynamic model can be divided into a system of M independent Equations (21).

(20) $$\vec{r_{i}}\left[n+T\right]=\vec{F}\left(\vec{r_{i}}\left[n\right],\Phi_{total}\left[T,n\right]\right)$$

(21) $$\{\begin{array}{c} r_{i}^{1}\left[n+T\right]=F_{1}\left(r_{i}^{1}\left[n\right],\Phi_{total}\left[T,n\right]\right)\\ \ldots \\ r_{i}^{M}\left[n+T\right]=F_{M}\left(r_{i}^{M}\left[n\right],\Phi_{total}\left[T,n\right]\right) \end{array}$$

Using this model, it must be guaranteed that no resource, during the planned operation time, goes below a certain acceptable limit $r_{th}^{j}$. These limits are calculated to gurantee the minimum survival of the node.

Each node will perform short measures (e.g., T=10 min) in order to estimate the medium value stochastic term. Using this information and the resource evaluation laws $F_{j}$, the node will calculate the resource state at the end of the planned operation time $T_{op}$ and if it is smaller than $r_{th}^{j}$ (for any resource) the CPO will be not accepted.

The final detail to be discussed are the definition of the resource evolution laws $F_{j}$. These laws must be defined before implementing the proposed algorithm using hardware technologies. They depend on the resource to be studied (for example battery charge presents an exponential evolution but memory usage follows a linear law) and should be transformed into Taylor’s series if the corresponding evolution law does not present a polynomic form (so it can be implemented using binary adders and multipliers).

Considering all previous details, it is possible to propose a schematic hardware implementation. The objective of this paper is not to describe a complete low-level hardware implementation which, even, could be performed using solid state devices and microelectronic techniques in order to improve the integration level. However, some important components and signals in order to understand the behaviour of the proposed hardware-supported algorithm are identified.

**Figure 14:**
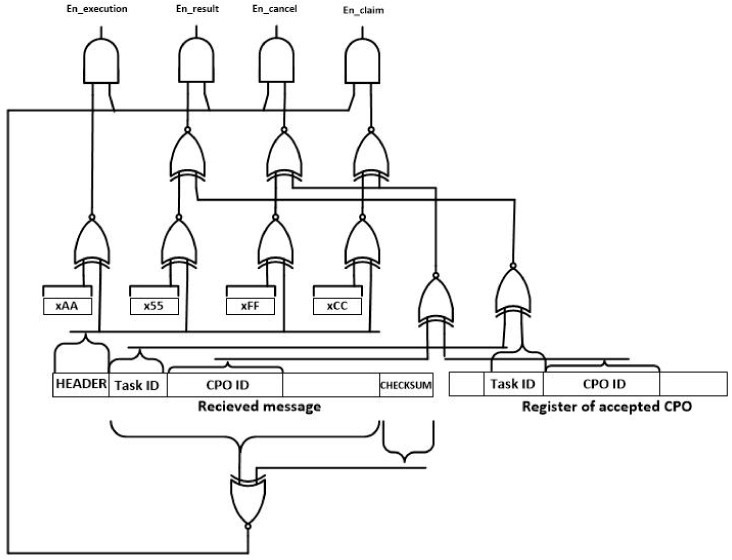
Enable signal generation.

The first module to be considered is the enable signal generation (see Figure 14). In this module and using the binary header of messages (se Section 3.2), the signals that activate the processing of the received message are generated. In order to do that and using XNOR logical gates, the header of the received message is compared to the four possible headers. Only the gate where both entries are equal produces a logical one at its output. Besides, if the received message is a result or a claim or cancellation message, the message must be referred to the CPO being currently executed. Besides, using a XNOR and a AND gate it is validated the correctness of the received message using the checksum. As a result, four enable signal are generated: “en_execution,” “en_result,” “en_cancel” and ”en_claim.” The signal associated to the received message type is set to high level.

**Figure 15:**
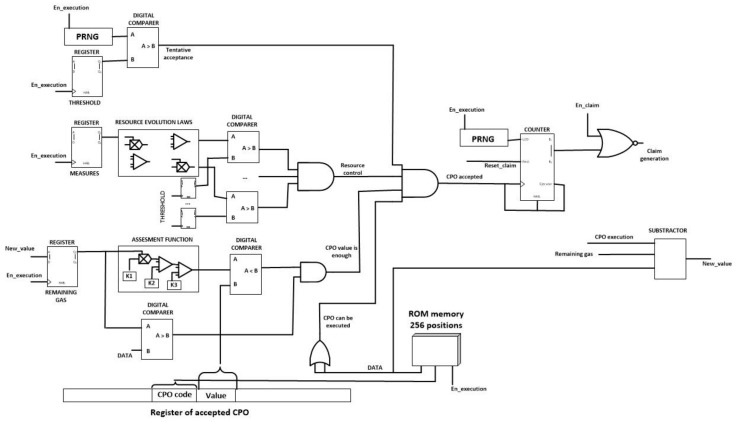
CPO evaluation module.

Figure 15 shows the CPO evaluation module. This circuit performs the self-management functions and the first steps in the choreographed execution of tasks. As can be seen, although in Figure 13 the proposed algorithm was showed in a sequential way, hardware technologies enable the parallel execution of the different verification phases, despite of the fact that smart nodes, usually, do not support parallel programming.

The unique important novelty in the proposed hardware implementation is the inclusion of a 256 position ROM memory. In this memory, only positions whose address corresponds with codes of CPO the node can perform have a non-empty content. The content of this memory is the amount of gas each CPO requires to be executed. In that way, doing only one query to the ROM memory two steps in the proposed algorithm are performed almost completely.

Moreover, in order to implement the entire algorithm using simple hardware technologies, Pseudorandom Number Generators (PRNG) might be constructed using XOR gates and Logical Shift Registers.

**Figure 16:**
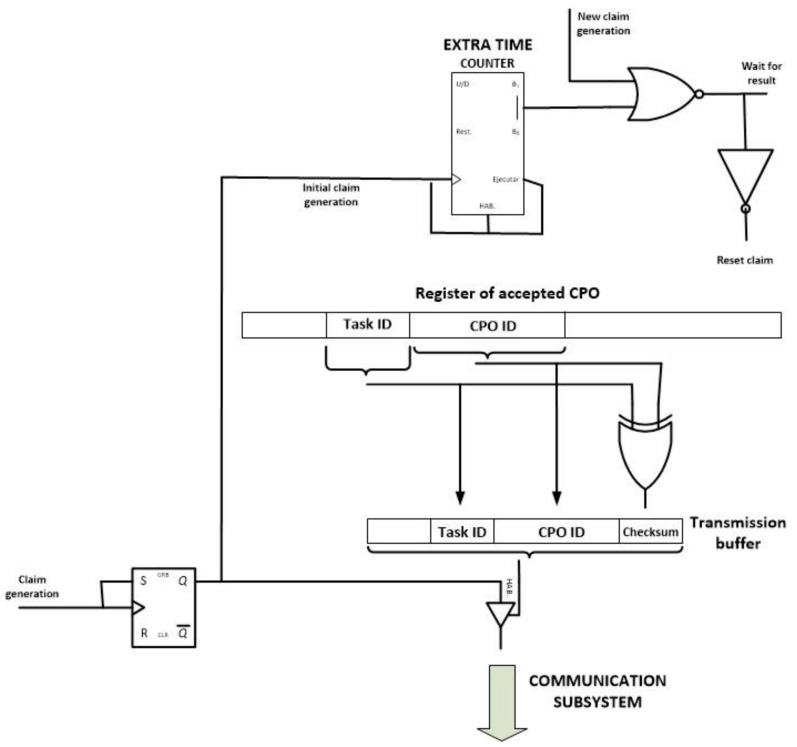
Claim module.

Once the “claim generation” signal is calculated, a second module starts its performance: the claim module (see Figure 16). This module generates and transmits the claim operation message (if needed) and performs the steps of the choreography algorithm that ensure that only one node in the WSN is responsible for executing a CPO.

The claim module includes a RS bistable which maintains the initial “claim generation signal” in its original value for an infinite time. This new signal (the “initial claim generation” signal) activates a counter which controls the extra waiting time for simultaneous claim messages. At the same time, a tristate buffer is enabled and the corresponding claim message is broadcasted. If during the extra time a new claim message is received, a reset signal is activated, so the last step in the CPO evaluation module is executed once more (a new random waiting time is initiated to solve the collision of claim messages). The inverse of this reset signal activates the last module of the proposed self-managed and choreographed task execution algorithm.

**Figure 17:**
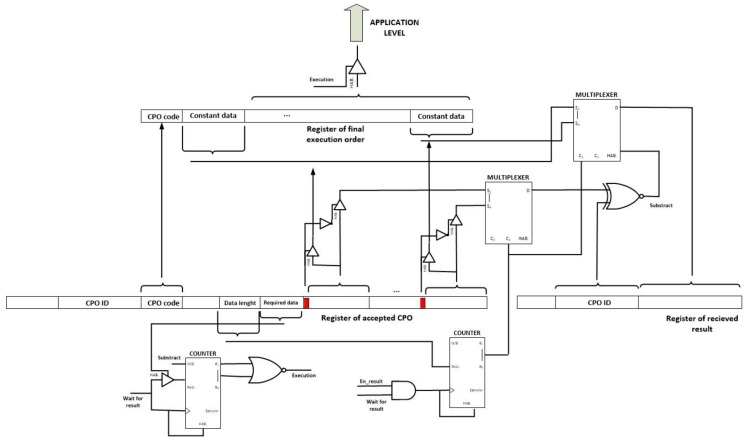
CPO execution module.

Thus and finally, the CPO execution module maintains accepted CPO in a waiting state, until the minimum number of input data are received and then the execution is finally performed (see Figure 17). In order to do that, a counter controls the number of constant data that have been received and compares the result with the required data specified in the execution order. Once both values are equal (the counter reaches the value zero), a tristate buffer sends the final CPO execution order to the application level.

Using also tristate buffers and the one bit flag that is included with each data in the CPO execution order, it is decided if the corresponding data is a constant value that can be included directly in the final execution order, or a cross-reference that must be replaced. Each time a result message is received, two multiplexers are employed to compare the CPO ID to the pending cross-references and, if success, replace the adequate reference with the received results.

Once the CPO is executed the result message can be generated and broadcasted using only one buffer. Besides, if the en_cancel signal gets activated at any time, all registers, counters and other digital circuits get reset using the appropriate pin of each element. In these both cases, no additional hardware implementation is required.

## 4. Experimental Validation

In this section, the performance of the proposed solution is evaluated. Two different types of experiments were carried out. The first type consisted of numerical simulations, employed to evaluate the correctness of the congestion control algorithm and other characteristics of the lightweight gateway behaviour. The second type was based on the ModelSim software, which enabled us to simulate the hardware implementation of the proposed self-management and choreography algorithm using the VHDL (Very High Speed Integrated Circuit Hardware Description Language) language. Using these ModelSim-based simulations we evaluated important low-level aspects of the proposed solution such as the processing delay.

In particular, four different experiments were planned and developed. The first three experiments were based on numerical simulations. The fourth one was developed using the ModelSim software.

The first experiment evaluated the performance of the proposed algorithm to obtain the value of vector $\vec{N}$, the number of devices in each server group. Ten different CPO were considered during this first experiment, so ten different server groups were defined. Each node was able to perform one or two different CPO. The total number of nodes in the simulation is one hundred (100). The specific composition of the WSN is showed in Table 1. Values in Table 1 represent the total number of nodes being able to perform the CPO indicated in the corresponding row and column. For example, two nodes can execute both the CPO#1 and CPO#3; but five nodes can only execute CPO#1.

The convergence speed and the final value for the $\vec{N}$ depend on the delegated task configuration and workflow (as said, the algorithm was specifically designed to depend on these parameters). Therefore, we must guarantee that the obtained results are coherent (they are detected, as maximum, as much devices as included in the WSN) and that final values are not oscillating.

The second experiment was designed to investigate if the QoS parameters in the WSN are guaranteed as desired. In particular, we evaluated if the proposed algorithms maintain the congestion factor and the network load below the specified limits. For this experiment, the same WSN described for the first experiment was employed. The medium service time was simulated to be one second, so results may be analysed in an easier way. Moreover, although as described in Section 3.3, the design process should start defining the expected loss probability in the execution system, in this experiment we are selecting a value for the utilization factor $\rho_{exp}=0.7$ regardless the associated loss probability.

The third experiment was planned to evaluate the success rate in the execution of delegated tasks. The simulation scenario was the same of the two previous experiments but in this case, besides, workflows of CPO were randomly generated to be executed using the proposed solution. The success rate depending on the capacity of queues was evaluated. In this case, besides, it is interesting to repeat the experiment for different network configurations. Thus, the third experiment was repeated for a network including 100 sensors nodes, 200 sensor nodes, etc. The composition was always maintained as described in Table 1.

Finally, the fourth experiment was developed using the ModelSim software. The proposed hardware implementation was described using the VHDL language in order to evaluate the mean processing delay (as there are random components which do not present a constant delay). In this scenario, only two resources were considered relevant: the memory capacity and the battery charge. The memory capacity was modelled using a decreasing linear function with the number of performed operations. The battery charge was modelled using a decreasing exponential function, written as a Taylor’s series. Additionally, the minimum value of gas was considered to be zero $v_{0}=0$ and the maximum value was defined to be 255, $v_{max}=255$. The total amount of gas at the initial time was 4096 units, $g_{0}=4096$. For this last experiment the total number of possible CPO, 256, were considered.

## 5. Results

In this Section, results of the experiments described in Section 4 are presented in an ordered way.

**Figure 18:**
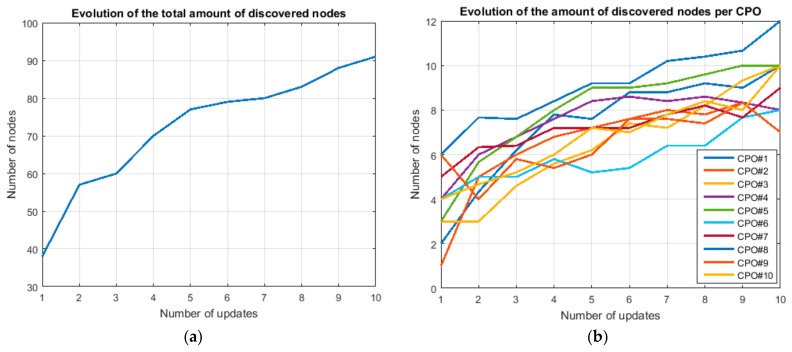
(**a**) Evolution of the total number of nodes (**b**) Evolution of the number of nodes per CPO.

Figure 18 shows the results for the first experiment. Figure 18a shows the result for the total number of nodes in the WSN and Figure 18b shows the result for each one of the ten available CPO. As can be seen, the evolution of the number of discovered nodes is globally increasing. After ten complete updates (several task executions that have not forced an update of the number of nodes could be also performed during this time), the total number of nodes has reached 90% of the final value. This result, with small variations, is also valid for each individual server group.

It is important to note that the evolution of $\vec{N}$ with the number of updates is not monotonous. There are fluctuations, because of the fact that a global improvement in the value of $\vec{N}$ might imply small partial deterioration in some values. The same effect appears when the new value of $\vec{N}$ is calculated as the average value of the two previous estimations.

However, as can be seen in Figure 18, calculated estimations are not oscillating, although they can fluctuate slightly. Moreover, with time, $\vec{N}$ converges to a stable value: in this case nodes are distributed uniformly among server groups as delegated tasks are generated randomly with a uniform distribution of CPO (as said, the algorithm is designed to distribute nodes among the different groups according to the real configuration of delegated tasks).

**Figure 19:**
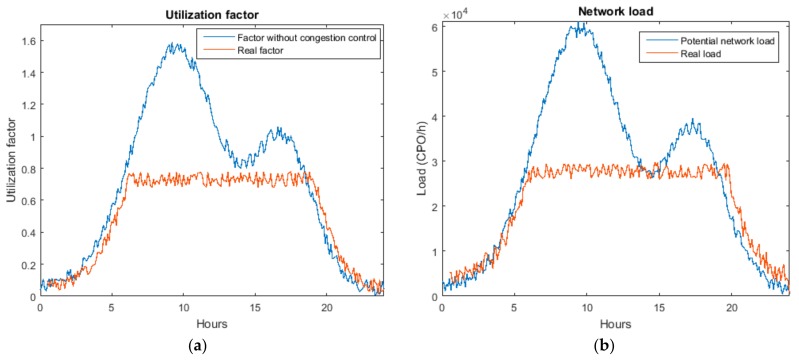
(**a**) Utilization factor (**b**) Network load.

During the second experiment, once it is proved the proposed algorithm to estimate the value of $\vec{N}$ presents the expected behaviour, we included in the simulation model the Erlang filter to evaluate the utilization factor and the network load. Figure 19 shows the obtained results. As the behaviour of the proposed filter is equal for all server groups, in this case, as can be seen, we are only studying one group. In that way, the performance of the proposed filter may be analysed in a clearer manner.

The operation of the WSN is simulated during twenty-four hours (24 h), considering a potential network load as showed in Figure 19b. This potential network load is directly obtained from the task delegation rate, which is configured to follow (during the day) a Gaussian-like evolution (with two peak of maximum use). This potential network load generates a potential utilization factor (see Figure 19a), where queues are supposed to be infinite.

Both presented figures are quite similar. The real utilization factor (or network load) follows the demand curve, while it allows guarantee the QoS of task executions. When demand goes above the maximum for which the WSN is designed, the Erlang filter prevents more CPOs from entering into the execution system than allowed per unit of time (exactly as planned). Small and continuous variations may appear due to the fact that the number of allowed CPO is an integer number but the network utilization factor is a real number. Besides, the effect of the slicing window included in the Erlang filter might also generate a high-frequency component.

It can be seen, also, that the execution system presents a delay in respect to the input task flow. The increasing (and decreasing) speed is lower, due to the impact of the filter that softens the variations that appear in the CPO flows.

**Figure 20:**
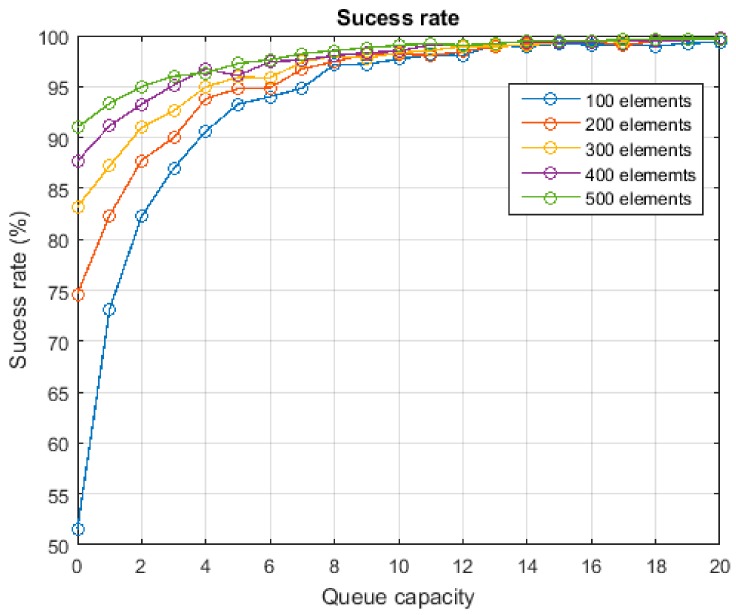
Evolution of the success rate.

Figure 20 shows the results of the third experiment. In this new experiment, once the WSN is guaranteed not to be congested, it is evaluated the success rate in task execution, when employing the proposed solution. In this experiment, we are only considering the CPOs (or tasks) that are accepted in the execution system. Those tasks that exceed the maximum allowed rate and were rejected in the Erlang filter are not included in the results of this experiment. All queues in the system are supposed to have the same capacity.

As can be seen, if no queue is considered, the success rate is around 52% when 100 sensors are considered in the WSN. As the number of nodes in the network grows, the success rate also goes up (for 500 nodes around 92%). For queues with a capacity of twenty (20) execution orders the success rate grows to almost 100%, independently from the number of nodes considered in the WSN. Actually, current computing systems may easily support queues with this capacity (considering the 256 different existing server groups, a total storage capacity of 5120 orders will be required).

These results are coherent with the proposed solution. It must be noted that, after being accepted and decomposed, CPO making up a task might be introduced in a queue if all nodes being able to execute that operation are busy. If any queue is full, the corresponding CPO will be rejected and, then, the entire task will be also rejected. In fact, although the maximum hourly delegation rate is not exceeded, it may appear delegation bursts which do exceed the capacity of the sensor network. This situation is more common as the queue capacity gets lower, which reduces dramatically the success rate as seen in Figure 20.

In the context of this experiment, where all values and structures present a uniform configuration (all queues have the same capacity, tasks are composed of random CPO following a uniform distribution, etc.), it can be proved that failures are practically due in full to the loss probability of the queue system (see Figure 10). The success rate will change depending on the composition of tasks and the queue capacity of each sever group. However, these first results show that the proposed solution is a valid mechanism to enable the self-managed and choreographed task execution in sensor networks.

**Figure 21:**
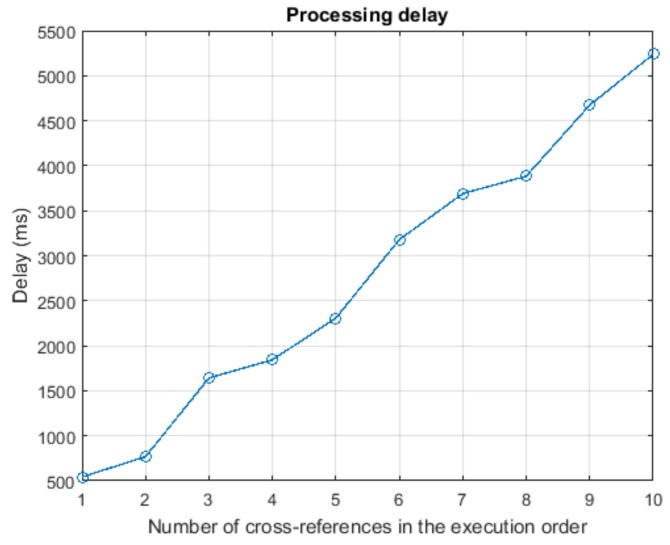
Processing delay.

Finally, for the fourth experiment, a simulated hardware implementation of the proposed algorithm was developed. Using this modelling and simulation tools the mean delay when processing an execution order is evaluated. For that, the delay between the reception of the execution order and the generation of the “execution” signal (see Figure 17) was evaluated. The experiment was repeated for different values of the field “required data” (see Figure 21).

As can be seen in Figure 21, the processing delay grows linearly with the number of cross-references. In order to guarantee this result and to avoid the WSN congestion (when exponential laws appear), the lightweight gateway must maintain the utilization factor below the unit (as proposed in this paper). In general, the minimum processing delay is slightly above 500 ms (including all random timers). From Figure 21, besides, it can be deducted that a workflow including ten (10) CPO requires around 6 seconds to be executed using the proposed solution.

ModelSim simulation may be also employed to obtain a footprint of the proposed solution. As the proposed algorithm is not a software solution, the footprint cannot be defined in terms of Flash Memory or SRAM. On the contrary and using the ModelSim capabilities, a good Key Performance Indicator (KPI) is the equivalent number of required logical gateways to implement the proposed hardware-supported algorithm.

Considering the maximum number of possible CPO and standard 16-bit counters, ModelSim software it is requested to generate the equivalent implementation using only logical gates. As different implementations can be selected, we obtained the mean values of all possible results. Considering all previous ideas, the calculated footprint for the proposed algorithm is 396 logical gates.

## 6. Conclusions

Wireless Sensor Networks (WSN) have evolved from a theoretical and research paradigm to be a real and practical technology. Actually, several new engineered systems such as Cyber-Physical Systems are based on WSN. Collaborative or choreographed task execution schemes fit in the most perfect way with the nature of sensor networks, as they enable the creation of flexible ad hoc resource pools being able to execute delegated tasks. Therefore, in this paper it is proposed a new solution for self-managed and choreographed task execution in sensor networks, which is adapted to resource-constraint devices and may be implemented in large WSN.

The solution includes a lightweight gateway where, using the Erlang’s queue theory, it is implemented a congestion control algorithm, so it is guaranteed the underlying WSN is always below the congestion level. In this context, choreography algorithms can operate without overloading the network because of the broadcast messages. This gateway also supports virtual nodes—named fictional nodes—employed to monitor and interact with the WSN in a transparent way.

Delegated tasks are filtered in the gateway and transformed into a set of execution orders, represented using a bit-oriented protocol and broadcast messages, so nodes may use a hardware supported algorithm to execute tasks in a choreographed way. In particular, the proposed algorithm includes both, a resource self-management module (where each node may decide if it has enough resources to execute a CPO) and a choreography module employed to decide which node (among which are able to execute the CPO) is finally in charge of the execution.

Results showed that the proposed solution reaches all the proposed objectives, controlling the congestion of the WSN and showing a linear evolution of time with the number of performed CPO.

## Figures and Tables

**Table 1:** Composition of the WSN during the first experiment.

	CPO#1	CPO#2	CPO#3	CPO#4	CPO#5	CPO#6	CPO#7	CPO#8	CPO#9	CPO#10
CPO#1	5	5	2							
CPO#2	5					4				
CPO#3	2				8					
CPO#4				10						
CPO#5			8					2		
CPO#6		4				4				
CPO#7								5	5	
CPO#8					2		5			5
CPO#9							5			4
CPO#10								5	4	1

## Abbreviations

The following abbreviations are used in this manuscript:

WSN	Wireless Sensor Networks
QoS	Quality of Service
CPO	Cyber-Physical Operation
